# Dietary Vitamin Intake and Blood Biomarkers in Relation to Muscle Activation in Amyotrophic Lateral Sclerosis: A Cross-Sectional Study

**DOI:** 10.3390/nu17213345

**Published:** 2025-10-24

**Authors:** Jose Enrique de la Rubia Ortí, Guillermo Bargues-Navarro, Jesús Privado, Rubén Menarques-Ramírez, Claudia Emmanuela Sanchis-Sanchis, Sandra Sancho-Castillo, Camila Peres Rubio, Luis Pardo-Marin, María Benlloch, Julio Martín-Ruiz

**Affiliations:** 1 Department of Basic Biomedical Sciences, Catholic University of Valencia, 46001 Valencia, Spain; joseenrique.delarubi@ucv.es; 2 Doctoral School, Catholic University of Valencia, 46001 Valencia, Spain; guillermo.bargues@mail.ucv.es (G.B.-N.); ce.sanchis@ucv.es (C.E.S.-S.); 3 Health Research Institute (IIS) La Fe, La Fe Polytechnic and University Hospital, 46026 Valencia, Spain; 4 Department of Methodology of Behavioral Sciences, Universidad Complutense de Madrid, 28040 Madrid, Spain; jesus.privado@pdi.ucm.es; 5 Nursing Department, Faculty of Health Sciences, University of Alicante, 03690 San Vicente del Raspeig, Spain; rmr23@alu.ua.es; 6 Department of Pathology, Catholic University of Valencia, 46001 Valencia, Spain; sandra.sancho@ucv.es; 7 Interdisciplinary Laboratory of Clinical Analysis, Regional Campus of International Excellence ‘Campus Mare Nostrum’, University of Murcia, Espinardo, 30100 Murcia, Spain; camila.peres@um.es (C.P.R.); lpm1@um.es (L.P.-M.); 8 Department of Health and Functional Evaluation, Faculty of Physical Activity and Sports Sciences, Catholic University of Valencia San Vicente Mártir, 46001 Valencia, Spain; julio.martin@ucv.es

**Keywords:** amyotrophic lateral sclerosis, vitamins, muscle activation, biomarker

## Abstract

**Background/Objectives**: Amyotrophic lateral sclerosis (ALS) is a neurodegenerative disease characterized by progressive loss of motor function, which affects mobility and leads to secondary complications, including altered dietary intake due to dysphagia, fatigue, and hypermetabolism, particularly affecting vitamin consumption, which are essential micronutrients for neuromuscular performance. The specific relationship between vitamin intake and muscle activation is not well understood in patients with ALS; thus, it is relevant to identify blood biomarkers that reflect muscle status. **Methods**: A cross-sectional study was conducted with 61 patients with bulbar- or spinal-onset ALS. The dietary intake of B vitamins (B1, B2, B6, B12, folate, and niacin); vitamins C, A, D, and E; and the B6/protein ratio were assessed using a seven-day dietary record and a Food Frequency Questionnaire. Blood concentrations of butyrylcholinesterase (BuChE), albumin, haptoglobin, C-reactive protein (CRP), and paraoxonase 1 (PON1) were determined. Basal muscle activation was measured using surface electromyography of the biceps brachii, triceps brachii, rectus femoris, and tibialis anterior muscles. Two confirmatory predictive models were developed to evaluate the effects of muscle damage and vitamin intake on muscle strength. **Results**: Arm muscle activation was negatively predicted by the B6/protein ratio (β = −0.33). Leg activation was positively predicted by vitamin B9 (β = 0.39) and B6/protein (β = 0.17) and negatively predicted by vitamin A (β = −0.24). For biomarkers, albumin (β = 0.18) and PON1 (β = 0.28) positively predicted activation. For legs, albumin predicted activation (β = 0.31), whereas BuChE and haptoglobin predicted negative activation (β = −0.32 and β = −0.15, respectively). **Conclusions**: Weak associations were observed in patients with ALS: vitamin B9 intake showed a modest association with leg activation, the B6/protein ratio exhibited inconsistent associations across muscle groups, and vitamin A showed a negative association with leg activation. Albumin demonstrated the most consistent association as a potential biomarker of muscle function. These findings are exploratory and require validation in larger, longitudinal studies.

## 1. Introduction

Amyotrophic lateral sclerosis (ALS) is a neurodegenerative disease characterized by progressive deterioration of the motor nervous system. It can present as a bulbar onset when upper motor neurons are initially affected or a spinal onset when lower motor neurons are compromised [1,2]. Clinically, the disease progresses rapidly, leading to severe disability due to the loss of motor unit potentials and, consequently, the inability to generate force [3,4]. As a result, mobility is impaired, and secondary complications arise that affect nutritional status, including dysphagia, meal-related fatigue, and hypermetabolism, which collectively compromise dietary intake [5,6]. The associated involuntary weight loss and decreased body mass index (BMI) result from a complex interplay of factors, including muscle atrophy (sarcopenia), increased resting energy expenditure, and progressive neurodegeneration, rather than solely from reduced caloric intake [7,8,9].

Therefore, the diet of patients becomes highly relevant, particularly due to the paradoxical hypermetabolism described in the early stages of the disease, where basal energy expenditure may increase by 200–1500 kcal above predicted values, primarily due to increased respiratory work and fasciculations [10,11].

Certain micronutrients, such as vitamins, play a key role in nutrient intake [12]. While some vitamins can be synthesized endogenously from precursors, produced by gut microbiota, or stored in body tissues, adequate dietary intake remains essential for maintaining optimal vitamin status, particularly for water-soluble vitamins and in conditions of increased metabolic demand [13,14,15]. Specific vitamins demonstrate neuroprotective mechanisms in ALS. Vitamin B12 (methylcobalamin) reduces oxidative stress and glutamate toxicity in motor neurons, with recent trials showing efficacy in early-stage ALS [16,17]. Vitamin D exhibits anti-inflammatory properties and supports muscle protein synthesis [18]. Vitamin E provides antioxidant protection and has been associated with reduced ALS risk [19]. Specific vitamin deficiencies lead to distinct consequences: Folate deficiency increases homocysteine levels, promoting motor neuron inflammation and degeneration [20]. In this sense, B12 deficiency increases homocysteine and neuroinflammation, vitamin D deficiency impairs muscle function, and vitamin E deficiency reduces antioxidant protection in motor neurons [21,22]. Moreover, antioxidant vitamins (E and D) modulate oxidative stress markers, including PON1 and haptoglobin [23,24]. The relationship between vitamin B6 intake and protein is especially important for muscle repair [25]. This makes vitamin deficiency particularly significant in neurodegenerative diseases with muscle deterioration, such as ALS.

To assess muscle damage in patients with ALS due to abnormal muscle activation, such as abrupt declines or increasing fasciculations, it is important to identify biomarkers linked to both muscle activity and dietary intake. Ideally, these should be quantifiable through blood analysis, as altered molecules related to neuromuscular junction function or peripheral muscle damage enter the circulatory system. In this context, serum quantification of molecules such as albumin [26], haptoglobin [27], C-reactive protein (CRP) [28], butyrylcholinesterase (BuChE) [29], and paraoxonase I (PON1) [30] may be of interest. However, there is still no evidence regarding the predictive power of these markers for muscle damage in diseases such as ALS.

This study aims to identify which vitamins predict muscle activation in ALS patients and determine the predictive value of blood biomarkers for muscle function. We hypothesize that specific vitamins will show distinct associations with muscle activation and that nutritional biomarkers will predict muscle function in ALS.

## 2. Materials and Methods

### 2.1. Subject Selection and Study Design

A cross-sectional selective design was carried out, in which participants were selected according to characteristics relevant to the study objectives.

A total of 61 patients with ALS were evaluated, with a mean age of 56.43 years (SD = 10.17 years). Of these, 65.6% were male. Bulbar-onset ALS was present in 16.4% of the cases and spinal onset in 83.6%. The mean time since diagnosis was 28.28 months (SD = 29.43 months), ranging from 2 to 146.

Patients were recruited through the main national ALS family and patient associations in Brazil. The study characteristics were provided to the patients via a patient information sheet, and those who agreed to participate signed an informed consent form. The selection criteria established in the study protocol were applied.

The inclusion criteria were as follows: men over 18 years; non-fertile women under 50 years or women aged 18–50 years; patients diagnosed with ALS at least six months prior to study initiation; and patients under riluzole treatment. The exclusion criteria were as follows: tracheostomy; invasive or noninvasive ventilation with positive pressure; participation in another clinical trial or in the 4 weeks prior to inclusion; evidence of dementia; dependence on alcohol or drug abuse; hepatitis B or C infection or HIV-positive status; renal impairment with creatinine levels twice above normal markers within 30 days prior to inclusion; and hepatic impairment with liver markers (ALT and AST) three times above normal levels. All participants provided informed consent before the study initiation. Patients were randomly recruited through national ALS associations as part of the clinical trial (NCT04654689).

The study was conducted at the IMED-UCV clinic in Valencia, Spain, between November 2021 and October 2022. The study protocol was developed in accordance with the ethical principles of the Declaration of Helsinki and was approved by the Ethics Committee of the University and Polytechnic Hospital La Fe of Valencia, Spain (code 2021-001989-38).

### 2.2. Measures

#### 2.2.1. Vitamins

Vitamin intake was estimated through dietary-nutritional anamnesis and information collection. To assess the initial food intake of patients, 24 h dietary recalls were administered over a seven-day period [31], along with a Food Frequency Questionnaire [32]. The combined use of both instruments provided detailed information on the consumption frequency of different food groups, including dairy products, vegetables, fruits, juices, nuts, meats, fish, seafood, eggs, tubers, rice, legumes, pasta, processed meats, snacks, pastries, cookies, chocolates, sugary beverages, and fermented and distilled alcoholic drinks. The one-week evaluation allowed for a more representative reflection of the participants’ habitual dietary patterns, minimizing the bias that could arise from analyzing a single day’s data [31].

The 24 h record consisted of a self-administered questionnaire [31]. In this record, patients noted the foods consumed daily, along with the ingredients used in meal preparation. They were also asked to specify the amounts consumed using common household measures (such as cup, portion, glass, tablespoon, slice, handful, plate, or ladle) or the exact weight of the food or beverage. To support this process, the participants were provided with a guide with equivalences between weight and commonly used measures [33]. The Food Frequency Questionnaire [32] included a table that patients were required to complete, specifying the frequency with which they usually consumed each food item.

#### 2.2.2. Diet and Eating Habits Assessment

The evaluation of dietary quality was conducted using the seven-day dietary record and the information obtained from the Food Frequency Questionnaire, processed through the DietoPro^®^ software (https://dietopro.com/, (accessed on 4 November 2021) PRO INI ONLINE version), a nutritional management tool developed in Valencia, Spain. This program allowed the creation of an individualized nutritional profile by calculating the daily averages of macronutrient and micronutrient intake. With regard to vitamins, daily estimates were obtained for B1, B2, B6, B12, folate, niacin, and vitamins C, A, D, and E, as well as the B6/protein ratio indicator, owing to its potential relevance to the study.

#### 2.2.3. Biomarker Determination

Blood samples were collected from all patients at 11 a.m. after an overnight fast. The samples were then centrifuged to separate the serum from the plasma. BuChE concentration was measured using the method reported by Tecles et al. [34]. PON1 activity was determined using 4-nitrophenyl acetate [35]. Albumin concentration was measured using commercial reagents (Beckman Coulter, Brea, CA, USA; OSR6102). Haptoglobin was determined with a colorimetric method (Tridelta Development Ltd., Kildare, Ireland) in an automatic analyzer (Cobas Mira Plus, ABX Diagnostica, Montpellier, France). A commercial solid-phase sandwich immunoassay (Tridelta Phase Range Canine C-reactive Protein Assay; Tridelta Development, Bray, Ireland) was used to determine CRP values, according to the manufacturer’s instructions.

All assays showed intra- and inter-assay imprecision below 10%. The low imprecision found in the assays justified the use of a single measurement rather than replicates.

#### 2.2.4. Muscle Activation

Electromyographic activity was recorded by a specialist experienced with ALS patient profiles. With the subject seated, the areas to be recorded were shaved and cleaned with alcohol to minimize impedance. Electrode placement was bilateral and longitudinal and on the muscle belly of the biceps brachii (BB), triceps brachii (TB), rectus femoris (RF), and tibialis anterior (TA), following SENIAM guidelines [36] and Criswell [37], using pediatric Lessa electrodes with a 3 cm diameter.

Recordings were performed using the Megawin ME6000-T8 device (Bittium Corporation, Oulu, Finland) at a sampling frequency of 1 kHz. Files were stored for 60 s with the subject in a static seated position to obtain precise activation values with greater correlation to patient muscle strength [38].

Subsequently, analog-to-digital conversion (12 bits; DAQCard-700; National Instrument, Austin, TX, USA) was performed using Biomonitor Megawin v.3.1 software, and files were stored on a hard drive for safekeeping and later analysis.

Signal processing was carried out with Matlab (R2025a) (Mathworks Inc., Natick, MA, USA) in three stages: First, signals were filtered using a fourth-order Butterworth band-pass filter admitting frequencies between 20 and 400 Hz. Next, Root Mean Square (RMS) was applied to normalize the signal to positive values. Finally, the signal was segmented by extracting the central 30 s to ensure stability, discarding initial adjustment values and final values potentially affected by fatigue.

Smoothed RMS averages were calculated for baseline values, and 10 peaks from each signal were recorded, arranged in descending order for fasciculation detection.

### 2.3. Data Analysis

First, the different descriptive statistics (mean and standard deviation) of the measures collected in this study were calculated. Second, Pearson’s correlations between the different measures were computed. These two analyses were performed using the statistical package SPSS V.23. Third, a Confirmatory Factor Analysis was conducted to examine whether the measures of muscle strength were grouped into common factors that reflected the shared variance of these measures. Fourth, two confirmatory predictive models were estimated to analyze the predictive role of muscle damage measures and vitamins on muscle strength. These last two confirmatory analyses were performed using the AMOS V.23 program [39].

To assess the fit of the data to the confirmatory models, two types of goodness-of-fit indices were employed. The first type was absolute indices, which were used to verify whether the theoretical model fit the empirical data collected. This included the χ^2^/df index [40], with values below 3 indicating a good fit; the Goodness-of-Fit Index (GFI) [41], with values > 0.95 considered a good fit; the Standardized Root Mean Square Residual (SRMR) [42]; the Root Mean Squared Error of Approximation (RMSEA) [43], with values < 0.08 indicating a good fit [44]; and the presence of < 5% of standardized residuals above 2.58 in absolute value [41,44]. The second type was incremental indices, which were used to compare the estimated model with the null model. This included the Normed Fit Index (NFI) [40] and the Comparative Fit Index (CFI) [45], with values > 0.95 indicating a good fit.

For this type of confirmatory model, 10 participants per indicator are recommended for factor analyses [46], although others recommend only five per indicator when the distribution is normal [44]. In our case, the second criterion was fulfilled, as we had 61 participants for 8–9 indicators of the different models tested (61/8 = 7.63; 61/9 = 6.78).

## 3. Results

### 3.1. Descriptive Statistics and Correlations

Table 1 presents the descriptive statistics (mean and standard deviation) for the different measures collected from patients with ALS. This table also reports Pearson’s correlations among the study variables. According to Cohen [47], the effect size of a correlation is considered small for values below 0.10, moderate up to 0.30, and large from 0.60 onwards. The effect size reflects the relevance of the correlation at the population level; thus, the larger the effect size, the greater the importance of the result at the population level, and consequently, the more generalizable the findings. Based on this criterion, we observed that vitamins showed negative correlations with muscle activation in the arms and positive correlations with muscle activation in the legs. In other words, higher vitamin levels were associated with lower muscle activation in the arms and higher muscle activation in the legs. Vitamin B6/protein exhibits the correlation with arm muscle activation (r = −0.24 to −0.34), whereas vitamin B9 shows the association with leg muscle activation (r = 0.23 to 0.36), followed by vitamin C (r = 0.16 to 0.31).

With respect to the correlation between muscle damage measures and muscle activation, it can be observed that the correlation trend is inverse, with positive correlations between muscle damage and muscle activation in the arms and negative correlations with muscle activation in the legs. However, this pattern did not hold for albumin, which showed positive correlations with both measures of muscle activation. For arm muscle activation, the measure with the strongest correlation was PON1 (r = 0.24–0.33), followed by albumin (r = 0.10–0.22). For leg muscle activation, the strongest correlations were found with albumin (r = 0.10–0.26), followed by BuChE (r = −0.21 to 0.00).

### 3.2. Confirmatory Factor Analysis

**Figure 1:**
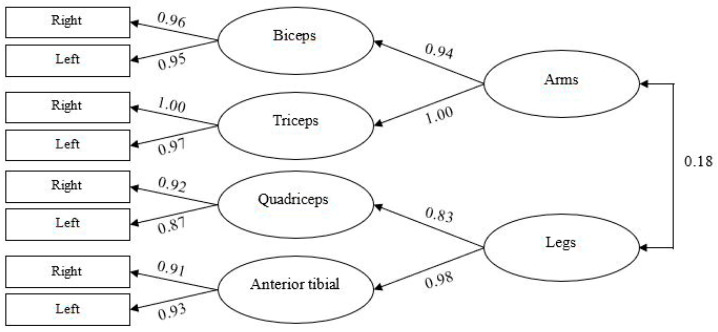
Confirmatory Factor Analysis for Muscle Activation.

A confirmatory factor model was estimated to capture the common variance among the different measures of muscle activation. Following Hair et al.’s [44] criterion, which requires that the indicators or variables of a latent construct have loadings of at least 0.40 to ensure a strong contribution to the underlying dimension, the final measures defining this construct are shown in Figure 1.

The model was estimated using the Unweighted Least Squares procedure, as the variables included in the model did not exhibit multivariate normality when assessed using the Bollen–Stine bootstrap [48] (*p* = 0.010). The goodness-of-fit indices indicated an adequate fit to the data (Table 2), and the factor loadings exceeded the minimum recommended threshold of |±0.40| [44], ranging from 0.83 to 1.00.

This result suggests that the different measures of muscle activation can be grouped into four first-order factors (biceps, triceps, quadriceps, and tibialis anterior), which can be combined into two second-order factors (arms and legs). Therefore, the activation measures displayed a hierarchical structure with two general factors explaining four more specific factors, which, in turn, accounted for the variance of the eight muscle strength measures. It should be noted that the correlation between the two second-order factors was relatively low (r = 0.18), which ruled out the existence of a general muscle activation factor.

### 3.3. Predictive Models

**Figure 2:**
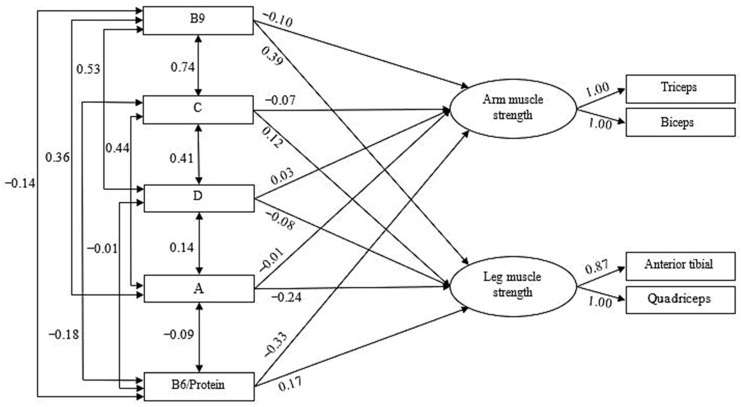
Predictive Model of Muscle Activation Based on Vitamins (B9, C, D, A and B6 vitamins).

Once the confirmatory model that grouped the measures of muscle activation into two second-order factors was estimated, the predictive role of vitamins and muscle damage measures on these two muscle activation factors was examined. Figure 2 shows the first of these predictive models (vitamins), estimated using the Maximum Likelihood procedure, given the multivariate normality estimated through the Bollen–Stine bootstrap (*p* = 0.104).

Table 2 presents the goodness-of-fit indices for the model. Not all vitamins were considered as predictors; vitamins with high intercorrelations (r ≥ |±0.80|) were removed from the model, since such high correlation values indicate general multicollinearity among the predictors, which prevents proper estimation of the confirmatory model [44]. As can be seen, the final model, once the highly correlated vitamins were eliminated, fits the data well according to these indices.

Regarding the predictors, the model incorporates the linear regression weights across the different measures. Following Cohen’s (1992) guidelines [47], an effect size of f^2^ = R^2^/(1 − R^2^) was considered small at 0.14, medium at 0.36, and large at 0.51. Based on this criterion, the vitamin-based predictive model indicated that arm muscle activation was predicted solely by the B6/protein ratio (β = −0.33), suggesting that higher values of this ratio were associated with reduced activation. For leg muscle activation, positive effects were observed for vitamin B9 (β = 0.39) and the B6/protein ratio (β = 0.17), whereas vitamin A showed a negative effect (β = −0.24). Thus, higher levels of B9 and B6/protein increased activation in the legs, whereas higher levels of vitamin A appeared to diminish it. Collectively, these predictors accounted for 12% of the variance in arm activation (R^2^ = 0.12) and 18% in leg activation (R^2^ = 0.18). We should clarify that when we talk about prediction, we are not referring to causality, since in our case, we did not use an experimental design. When we talk about prediction, we are referring to the fact that we theoretically assume that there are predictors and criteria (dependent variables) that are related in such a way that the former predicts the latter.

**Figure 3:**
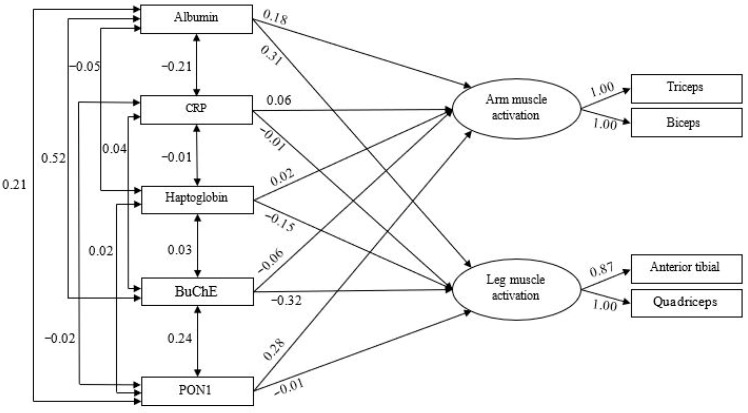
Predictive Model of Muscle Activation Based on Muscle Damage. BuChE: butyrylcholinesterase; PON 1: paraoxonase 1; CRP: C-reactive protein.

The predictive model for muscle damage is presented in Figure 3 and was estimated using the Maximum Likelihood method, with multivariate normality confirmed through the Bollen–Stine bootstrap (*p* = 0.488). The goodness-of-fit indices for this model (Table 2) suggest an adequate fit to the data. In this case, arm muscle activation was explained to a modest degree (11%, R^2^ = 0.11), with albumin (β = 0.18) and PON1 (β = 0.28) as significant positive predictors. For the legs, 13% of the variance (R^2^ = 0.13) was explained, with albumin (β = 0.31) positively associated with activation, whereas BuChE (β = −0.32) and haptoglobin (β = −0.15) were negatively associated.

## 4. Discussion

Among all micronutrients, vitamins stand out for their regulatory functions, as they participate in cellular metabolism with significant antioxidant activity [49]. B-group vitamins are key factors in the development of the nervous system [50], and their deficiency has been pathologically linked to neurodegenerative diseases such as Alzheimer’s, Parkinson’s, and ALS [51]. Specifically, in ALS, low-dose supplementation of vitamin B has been associated with improvements in the quality of life [52]. In our predictive model, which aimed to assess which vitamins ingested by patients with ALS could predict muscle activation—a process linked to the capacity to generate force—we found that, among all B vitamins, B9 significantly predicted activation, but only in the leg muscles (tibialis anterior and quadriceps). These findings reinforce the role of vitamin B9 in muscle physiology, particularly through its contribution to muscle blood flow [53], which explains its importance in muscle activation [54].

Interestingly, in this case, which was limited to arm muscles, the B6/protein ratio emerged as a negative predictor of muscle activation. It is important to highlight that although vitamin B6 and proteins are essential nutrients for muscle function, inadequate intake may have negative consequences, especially when consumed in an unbalanced manner or in amounts exceeding the recommended levels. For example, because vitamin B6 is essential for amino acid metabolism, increased protein intake requires greater B6 availability to maintain metabolic homeostasis [55]. If B6 intake does not increase proportionally with protein consumption, a functional vitamin B6 deficiency may develop, impairing neurotransmitter synthesis, energy efficiency, and muscle regeneration [56]. In our case, the negative effect would stem from an imbalance in which elevated B6 intake relative to protein increases B6-related toxicity in the muscle, previously described as leading to sensory neuropathy accompanied by weakness and fasciculations, indirectly compromising muscle function [57,58,59]. However, this negative relationship was observed only for arm activation in the present model. In the legs, the opposite occurred: higher B6/protein ratios were associated with increased muscle activation. These contradictory results, depending on the muscle group analyzed, underscore the need for further investigation into the relationship between the B6/protein ratios and muscle activity.

However, it is noteworthy that our model did not identify vitamin C or vitamin D intake as predictors of muscle activation in any of the analyzed muscle groups of the arms or legs. This is surprising, given the well-documented importance of both molecules in muscle function and strength. Ascorbic acid (vitamin C) is a potent antioxidant, and its intake may be particularly relevant to neurodegenerative processes [60]. At the muscular level, vitamin C deficiency results in fatigue and muscle weakness due to alterations in energy metabolism and carnitine synthesis [61]. However, muscle tissue is highly sensitive to vitamin C levels, and daily intakes of ~70 mg are sufficient to saturate the muscle, whereas lower levels (e.g., 30 mg/day) are clearly insufficient [61], which may help explain our findings. Furthermore, regarding its direct impact on ALS, while the administration of other antioxidants has shown protective effects against the disease, vitamin C intake has not been found to reduce the risk of developing ALS [62].

Vitamin D, in turn, is involved in several processes related to the central nervous system (CNS), the peripheral nervous system (PNS), and skeletal muscle, playing a key role in the development of neurodegenerative diseases [63]. However, its relationship with ALS remains contradictory; some studies have reported beneficial functional effects of supplementation [64], whereas others have found negative outcomes in patients with ALS [65].

Finally, it is important to highlight the negative role attributed to vitamin A intake in relation to leg muscle activation. Evidence has shown the potential toxicity of this vitamin at the muscular level. Specifically, excessive intake has been associated with adverse effects on muscle tissue and function, including stiffness, muscle fatigue, accelerated protein degradation, and oxidative damage to the skeletal muscle [66,67].

With regard to biomarkers that may assist in the clinical assessment of muscle function alterations or disease progression in patients with ALS, albumin, CRP, haptoglobin, BuChE, and PON1 have all been linked to muscle activity or damage and are considered to play significant roles [27,30,68,69,70]. However, in our predictive model, not all factors appeared to predict muscle function. Specifically, albumin and BuChE levels were found to positively and negatively predict leg muscle activation, respectively, with albumin also emerging as a weaker predictor of arm muscle activation (β = 0.18). These findings support albumin as a reliable indirect biomarker of nutritional status and muscle mass [59]. In contrast, the role of BuChE warrants further investigation, as despite its association with improvements following nutritional interventions in conditions such as multiple sclerosis [71], its peripheral function remains controversial. In addition, PON1 activity positively predicted arm muscle activation, such that higher enzyme activity was associated with improved muscle function, thereby confirming its antioxidant role in conditions involving muscle damage [30]. In contrast, haptoglobin negatively predicted muscle activation, which may be explained by its protective role against muscle injury, reducing oxidative stress and modulating inflammation derived from hemoglobin release after muscle damage [27] while simultaneously serving as an indirect marker of muscle injury and the associated inflammatory response in pathologies such as muscular dystrophy or inflammatory myopathies [72]. Interestingly, despite its activity, CRP did not appear to predict muscle function, according to our results. In this sense, although CRP is a useful marker of systemic inflammation, it shows weak or modest correlations with muscle strength or mass in chronic diseases or aging [73]. That is, although an association exists, it may lack the sensitivity needed to detect localized or segmental muscle damage, such as that assessed in the arm versus the leg.

On the other hand, biomarkers such as haptoglobin have been shown to play a direct role in protecting against oxidative stress and muscle atrophy in animal models [27], likely reflecting mechanisms that are more specific to the muscle itself, which could explain their higher predictive value in our study. Moreover, although some studies in ALS have observed that CRP is associated with overall functional progression (ALSFRS-R) and survival [74], these findings do not necessarily capture localized functional muscle changes or the muscle activation patterns identified in our analysis.

Overall, while albumin and PON1 were associated with increased muscle activation in the arms, leg muscle function was positively predicted by albumin and negatively predicted by BuChE and haptoglobin levels.

In conclusion, in light of the study’s objectives, the predictive models indicated that vitamin B9 intake positively influenced leg muscle activation in patients with ALS. Conversely, the relationship between vitamin B6 and protein intake showed mixed results: both were positively linked to leg muscle activation but negatively linked to arm muscle activation. Among the blood biomarkers potentially reflecting muscle activity status, serum albumin was notable for its positive association with muscle activation in both the legs and the arms.

Despite these findings, this study has some limitations. The most notable limitation is the reduced sample size, which clearly undermines the statistical power of the contrast. Although ALS is classified as a rare disease owing to its low prevalence, the cohort analyzed remains small, highlighting the importance of replicating the analysis in a larger population. Furthermore, the cross-sectional design does not allow for causal inference between vitamin intake and muscle activation or biomarkers. Our findings describe associations and should be interpreted as exploratory rather than predictive, and no causal conclusions can be drawn from the observed relationships. Another significant limitation of this study is the reliance on dietary intake questionnaires (24 h recalls and Food Frequency Questionnaire) to assess vitamin status, rather than direct biochemical measurement of circulating vitamin concentrations. While dietary assessment provides valuable information about nutrient intake patterns, it does not account for individual variability in vitamin absorption, bioavailability, or metabolic alterations, which are particularly relevant in ALS. Self-reported dietary methods are also prone to recall bias, memory errors, and subjectivity, especially in ALS patients who may experience speech difficulties, fatigue, or cognitive impairment. Direct quantification of serum vitamin levels (e.g., vitamins B6 and B9) through validated biochemical methods such as ELISA or HPLC would have provided a more accurate and objective assessment of vitamin status. Future studies should prioritize the measurement of circulating vitamin biomarkers to more reliably establish their relationship with muscle activation and disease progression in ALS. Another limitation is that biomarkers were measured at a single timepoint. Although the biomarkers assessed (albumin, CRP, BuChE, PON1, and haptoglobin) exhibit relatively low intra-individual variability, a single measurement does not fully capture the dynamic nature of inflammatory, oxidative, and metabolic processes that evolve throughout ALS disease progression. Longitudinal biomarker assessments would provide more comprehensive insights into the temporal relationships between nutritional status, inflammation, and muscle function. Moreover, the biomarker panel did not include additional markers of neuronal degeneration (e.g., neurofilament light chain), muscle damage (e.g., creatine kinase), or specific nutritional deficiencies (e.g., homocysteine), which would have enriched the interpretation of muscle activation in ALS. Future research should incorporate a broader multi-biomarker approach. Finally, important potential confounders were not systematically controlled in the analyses. Variables such as disease stage (assessed by ALSFRS-R score or disease duration), body mass index, level of physical activity, and use of vitamins or nutritional supplements may influence both vitamin intake and muscle activation. The absence of adjustment for these factors limits our ability to isolate the independent effects of dietary vitamins on muscle function and may introduce residual confounding.

## 5. Conclusions

Considering the study’s objectives, the analyses revealed weak associations between dietary vitamin intake and muscle activation in patients with ALS. Specifically, vitamin B9 intake showed a modest positive association with leg muscle activation, while the B6/protein ratio exhibited inconsistent associations: a weak positive association with leg muscle activation but a negative association with arm muscle activation. Regarding blood-based biomarkers that may reflect the status of muscle activity, serum albumin stood out, as it positively predicted muscle activation in both legs and arms. These exploratory findings require verification through longitudinal studies with larger sample sizes and appropriate control for confounding factors before any causal or predictive interpretations can be established.

Despite these findings, this study has some limitations. The most notable limitation is the reduced sample size. Although ALS is classified as a rare disease owing to its low prevalence, the cohort analyzed remains small, highlighting the importance of replicating the analysis in a larger population. Furthermore, as this is a cross-sectional study, it would be valuable to examine these results in relation to patient age and disease duration since diagnosis in future studies.

## Figures and Tables

**Table 1:** Descriptive Statistics and Pearson Correlations.

	1	2	3	4	5	6	7	8	9	10	11	12	13	14	15	16	17	18	19	20	21	22	23	24
1. Vitamin B1 (mg)	1.00																							
2. Vitamin B2 (mg)	0.99	1.00																						
3. Vitamin B6 (mg)	0.75	0.75	1.00																					
4. Vitamin B12 (µg)	0.99	0.98	0.78	1.00																				
5. Vitamin B9 (µg)	0.53	0.53	0.40	0.55	1.00																			
6. Vitamin B3 (mg)	0.17	0.18	0.78	0.24	0.12	1.00																		
7. Vitamin C (mg)	0.40	0.40	0.18	0.41	0.74	−0.10	1.00																	
8. Vitamin A (µg)	0.10	0.12	−0.01	0.14	0.36	−0.10	0.44	1.00																
9. Vitamin D (µg)	0.98	0.97	0.71	0.98	0.53	0.14	0.41	0.14	1.00															
10. Vitamin E (mg)	0.98	0.98	0.71	0.98	0.61	0.13	0.48	0.17	0.97	1.00														
11. Vitamin B6/Protein	−0.02	−0.02	−0.03	−0.02	−0.14	−0.03	−0.18	−0.09	−0.01	−0.04	1.00													
12. Albumin (g/dL)	−0.20	−0.20	−0.12	−0.21	0.06	0.00	0.06	0.07	−0.18	−0.18	−0.15	1.00												
13. CRP (mg/L)	−0.08	−0.07	−0.02	−0.09	−0.07	0.05	−0.06	−0.02	−0.06	−0.11	−0.05	−0.21	1.00											
14. Haptoglobin (mg/dL)	−0.09	−0.08	−0.34	−0.12	0.02	−0.42	0.22	0.10	−0.05	−0.03	−0.02	−0.05	−0.01	1.00										
15. BuChE (UI/L)	−0.11	−0.12	0.04	−0.15	−0.04	0.17	−0.18	−0.15	−0.14	−0.14	−0.14	0.52	0.04	0.03	1.00									
16. PON 1 (UI/L)	−0.22	−0.22	−0.18	−0.21	−0.25	−0.08	−0.17	−0.14	−0.19	−0.20	0.01	0.21	−0.02	0.02	0.24	1.00								
17. Right Biceps Muscle Activation	**−0.13**	**−0.12**	**−0.12**	**−0.12**	**−0.10**	−0.06	**−0.10**	**−0.10**	−0.08	**−0.13**	**−0.32**	**0.10**	−0.03	0.03	0.00	**0.24**	1.00							
18. Left Biceps Muscle Activation	**−0.13**	**−0.12**	**−0.12**	**−0.12**	**−0.14**	−0.06	**−0.11**	−0.01	−0.08	**−0.13**	**−0.32**	**0.22**	0.02	0.03	**0.14**	**0.29**	0.91	1.00						
19. Right Triceps Muscle Activation	−0.05	−0.05	−0.07	−0.05	−0.04	−0.06	0.02	**−0.10**	0.00	−0.05	**−0.26**	**0.17**	−0.01	0.00	0.06	**0.28**	0.92	0.86	1.00					
20. Left Triceps Muscle Activation	−0.04	−0.04	−0.05	−0.03	−0.04	−0.05	−0.05	−0.03	0.02	−0.03	**−0.24**	**0.21**	0.03	−0.02	**0.12**	**0.33**	0.86	0.94	0.90	1.00				
21. Right Rectus Femoris Muscle Activation	**0.10**	0.07	0.09	**0.11**	**0.32**	0.03	**0.25**	**−0.14**	**0.12**	**0.13**	**0.10**	**0.10**	−0.06	**−0.17**	**−0.21**	−0.04	0.11	0.03	0.22	0.12	1.00			
22. Left Rectus Femoris Muscle Activation	**0.11**	0.09	0.09	**0.13**	**0.27**	0.03	**0.16**	−0.02	**0.14**	**0.14**	**0.11**	**0.14**	−0.08	**−0.21**	**−0.14**	−0.02	0.13	0.07	0.23	0.13	0.80	1.00		
23. Right Anterior Tibialis Muscle Activation	0.09	0.06	0.02	0.07	**0.23**	−0.06	**0.18**	−0.05	0.09	**0.10**	0.09	**0.15**	**−0.10**	−0.07	−0.06	−0.03	0.16	0.11	0.25	0.16	0.70	0.59	1.00	
24. Left Anterior Tibialis Muscle Activation	**0.10**	0.08	−0.01	**0.10**	**0.36**	**−0.12**	**0.31**	**0.11**	**0.13**	**0.13**	**0.10**	**0.26**	**−0.10**	0.00	−0.07	0.03	0.11	0.09	0.21	0.14	0.68	0.70	0.85	1.00
Mean	7.99	9.08	14.26	16.04	318.03	75.49	174.55	1133.13	14.59	20.04	5.7	4.21	6.58	371.53	4.66	3.08	3.34	3.31	3.48	3.38	3.87	3.89	2.92	2.75
SD	48.33	48.55	64.3	54.83	157.51	277.95	114.23	1013.89	48.1	50.02	41.09	0.24	5.93	118.01	1.41	0.81	1.35	1.35	1.25	1.29	1.48	1.32	1.65	1.66

Correlations ≥ |±0.26| are statistically significant. Correlations between vitamins, muscle damage, and muscle activation with at least a small effect size (≥|±0.10|) are shown in bold. BuChE: butyrylcholinesterase; PON 1: paraoxonase 1; CRP: C-reactive protein.

**Table 2:** Goodness-of-fit indices for the Confirmatory Factor Analysis and Predictive Models.

Models	*χ* ^2^ */df*	GFI	NFI	CFI	TLI	RMSEA	SRMR	Residues ≥ |±2.58|
Confirmatory Factor Analysis	0.81	0.996	0.993				0.043	0.00%
Predictive Model: Vitamins	1.58	0.929	0.955	0.982	0.954	0.099	0.072	0.00%
Predictive Model: Muscle Damage	1.04	0.952	0.966	0.999	0.997	0.025	0.047	0.00%

CFI: Parsimony Comparative Fit Index; GFI: Goodness-of-Fit Index; NFI: Normed Fit Index; RMSEA: Root Mean Squared Errors; SRMR: Standardized Root Mean Square; TLI: Tucker–Lewis Index.

## Data Availability

The data that support the findings of this study are available on request from the corresponding author. The data are not publicly available due to privacy or ethical restrictions.

## Abbreviations

The following abbreviations are used in this manuscript:

ALS	Amyotrophic Lateral Sclerosis
BuChE	Butyrylcholinesterase
CFI	Parsimony Comparative Fit Index
CRP	C-Reactive Protein
GFI	Goodness-of-Fit Index
NFI	Normed Fit Index
PON1	Paraoxonase 1
RMS	Root Mean Square
RMSEA	Root Mean Squared Errors
SRMR	Standardized Root Mean Square
TLI	Tuker-Lewiss Index
